# Comparison of Patients’ Phenotypes, Guideline-Directed Recommendations Compliance and Rates of Cardiotoxicity between Caribbean and United States Cardio-oncology Programs

**DOI:** 10.5334/gh.1153

**Published:** 2022-09-20

**Authors:** Pamela Piña, Amparo Taveras, Amir Khan, Justin Coyle, Victor Bueno, Nishit Shah, Luis Cuello, Santiago Collado, Ann Mauer, Sorin Danciu, Cesar J. Herrera

**Affiliations:** 1 Department of Cardiology, CEDIMAT Cardiovascular Center, Santo Domingo, Dominican Republic; 2 Section of Cardiology, Advocate Illinois Masonic Medical Center, Chicago, US

**Keywords:** Cardio-Oncology

## Abstract

**Background:**

Little is known about the characteristics of oncological patients, cancer therapy-induced cardiotoxicity, and guidelines-directed interventions in the Caribbean; analysis of cardio-oncology services may shed light on this and clarify links between ethnicity, cultural, and local socioeconomic factors.

**Objectives:**

This study compared patients’ phenotypes, adherence to guidelines recommendations, and patterns of cardiotoxicity between two cardio-oncology programs: one in the Dominican Republic (DR) and the other in Chicago IL, United States (US).

**Methods:**

Patients being considered for or treated with potentially cardiotoxic drugs were followed before, during, and after chemotherapy through both cardio-oncology clinics, where we recorded and compared clinical, demographic, and echocardiographic data.

**Results:**

We studied 597 consecutive patients, 330 (55%) from the DR and 267 (45%) from the US. DR vs. US mean age 55± 13/52 ± 13 years; female 77/87% (p < 0.001); breast cancer 57/73% (p < 0.001); treated with anthracyclines + taxanes 47/40% (p = 0.151); monoclonal antibodies + taxanes or platins 37/45% (p < 0.001). Cardiotoxicity DR vs. US occurred in 15/7% (p = 0.001); multivariate logistic regression (OR 2.29; 95% CI, 1.31–3.99; p < 0.005) did not identify age >60, HTN, DM, BMI, tobacco or chemotherapy as predictors. Compliance with ASCO guidelines was similar among both cohorts.

**Conclusion:**

Compared to the US cohort, the Caribbean cohort of cancer patients has similar rates of CV risk factors but a higher likelihood of developing drug-induced LV dysfunction. Programs’ compliance with ASCO guidelines was equivalent. While further research is needed to ascertain regional variations of cardiotoxicity, these findings underline the relevance of cardio-oncology services in nations with limited resources and high CV risk.

## Introduction

The longevity of cancer patients is increasing owing to early detection and improvements in antineoplastic therapies. By January 2019, 16.9 million Americans with a history of cancer survived in the United States (US) [1], and it is estimated that there will be more than 22 million cancer survivors worldwide by 2030 [1,2]. Yet cardiovascular (CV) mortality in cancer survivors remain high, even though both conditions share similar risk factors. In addition, many antineoplastic agents are fraught with cardiotoxic potential. This paradox has enhanced awareness of the link between cardiac and oncological disease and the ensuing need for integration of cardio-oncology services.

The Dominican Republic (DR), the largest Caribbean nation after Cuba with a population of nearly 11 million inhabitants, reported 17,000 new cancer cases in 2018, the most frequent being breast, prostate, and colorectal [3]. According to the World Health Organization, 25% of breast cancer diagnoses in the DR are for women under 35 years of age, a pattern that highlights an important psychosocial and financial burden in a country where 94% of the population is younger than 65 years old [4]. A recent survey on the prevalence of CV risks in the DR (ENPREFAR-HAS) reported alarming figures: 31% of adults have hypertension, 29% are affected by obesity, 12% self-report as regular smokers, and the prevalence of diabetes approached 6% [5]. However, there are no data on the regional CV profile of oncological patients in the Caribbean, nor are there systematically applied protocols designed for the prevention and monitoring during and after treatment. Structuring registries and cardio-oncology services in Latin America are a challenge, but once established, they may offer an opportunity to understand better the relationship between ethnicity, socioeconomic factors, and outcomes in this rapidly evolving field.

We have recently established a local registry that intends to identify the socio-demographic profile, the prevalence of CV risk factors, echocardiographic, and biomarkers utilization patterns in a third-level institution in DR. The goal of our study was to compare patterns of cardiotoxicity (CTx), the prevalence of CV risk factors, and American Society of Clinical Oncology (ASCO) guideline adherence between an oncologic program performed in a limited resources nation (DR) and a program in a high resources country (US).

## Methods

This prospective observational study was conducted at CEDIMAT Cardiovascular Center in the Dominican Republic and Advocate Illinois Masonic Medical Center in Chicago, US. All newly diagnosed cancer patients >18 years of age being considered for or treated with potentially cardiotoxic drugs were included and followed during a 37-months period in the DR and 43 months in the US. A total of 597 consecutive patients were included, 330 (55%) from the DR and 267 (45%) from the US. Patients were evaluated before, during, and after chemotherapy through both cardio-oncology clinics, where symptoms and echocardiographic data were recorded. CTx was defined as a left ventricle ejection fraction (LVEF) reduction >10 % to a value < 53% from the initial assessment or an absolute global longitudinal strain (GLS) reduction >15%, and asymptomatic patients with these findings were diagnosed with subclinical cardiotoxicity [6]. The analysis of other echocardiographic parameters beyond LV contractility and deformation is not within this investigation’s scope, nor other toxicities related to chemotherapy besides LV dysfunction.

The data collection protocol included the presence of obesity (Body mass index [BMI] ˃ 30), hypertension (HTN), diabetes mellitus (DM), dyslipidemia, tobacco use, previously known CAD, type of cancer, and antineoplastic treatment regimen, as well as documentation of LVEF, GLS, NT-proBNP and troponin levels during each evaluation. Notably, echocardiograms were performed at each institution by the same group of physicians; all parameters previously detailed were measured similarly in all patients in both countries. None of the investigators or echo readers participated in the patients’ initial care; treating physicians, though they were made aware of the details of the registry, received no instructions from investigators. The study protocol followed the ethical guidelines of the 1975 Declaration of Helsinki as reflected in the a priori approval by the Institution’s Human Research Committee.

Within- and between-group comparisons of changes in cardiac parameters were obtained and analyzed with paired *t*-tests using Epi Info version 7 software. Results were expressed as mean ± SD or median, and a p-value of <0.005 was considered statistically significant. Multivariate Cox regression analysis was performed to determine independent predictors for CTx; model results were presented with odds ratio (OR), 95% confidence interval (CI), and p values.

## Results

From February 2016 to September 2019 a total of 597 consecutive patients were included, 330 (55%) from the DR cohort and 267 (45%) from the US cohort, who constituted the study group. Most referring providers (DR vs US 90/85%) were staff members of participating hospitals. Female gender was the most prevalent [254 (77%)/232 (87%)] with a mean age of 54 years. Breast cancer was the most common amongst both cohorts. Risk factors for CTx and treatment combination of potentially cardiotoxic drugs were also analyzed, as shown in Table 1.

**Table 2:** Baseline demographic and clinical characteristics of both cohorts.


	DR (n = 330)	US (n = 267)	TOTAL (n = 597)	*p*

**Mean age – (yrs.)**	55 ± 13	52 ± 13	54 ± 13	

**Female gender – n (%)**	254 (77)	232 (87)	486 (81)	p < 0.001

**Type of cancer – n (%)**				

Breast cancer	189 (57)	195 (73)	384 (64)	p < 0.001

Colon cancer	36 (11)	0	36 (6)	p < 0.001

Non-Hodgkin’s Lymphoma	11 (3)	22 (8)	33 (6)	p < 0.005

Lung cancer	20 (6)	3 (1)	23 (4)	p < 0.005

Hodgkin’s Lymphoma	10 (3)	11 (4)	21 (4)	p = 0.072

**Cardiovascular risk factors – n (%)**				

HTN	166 (50)	98 (37)	264 (44)	p < 0.005

DM	44 (13)	44 (16)	88 (15)	p = 0.163

Smoking	53 (16)	25 (9)	78 (13)	p < 0.001

BMI ≥ 30	108 (33)	87 (33)	195 (33)	p = 0.524

DLP	52 (16)	80 (30)	132 (22)	p < 0.005

≥ 2 risk factors	131 (39	94 (35)	225 (38)	p = 0.142

**Treatment – n (%)**				

Cardioprotective drugs	160 (48)	114 (43)	274 (92)	p = 0.095

Radiotherapy	145 (44)	162 (61)	283 (47%)	p = 0.001

Treated with anthracyclines + taxanes	151 (47)	106 (40)	257 (87)	p = 0.151

Treated with monoclonal antibodies + taxanes or platins	124 (37)	122 (45)	246 (83)	p < 0.001

**Developed cardiotoxicity – n (%)**	51 (15)	20 (7)	71 (24)	p < 0.001


BMI: body mass index, DLP: dyslipidemia, DM: diabetes mellitus, DR: Dominican Republic, HTN: hypertension, US: United States.

**Figure 1:**
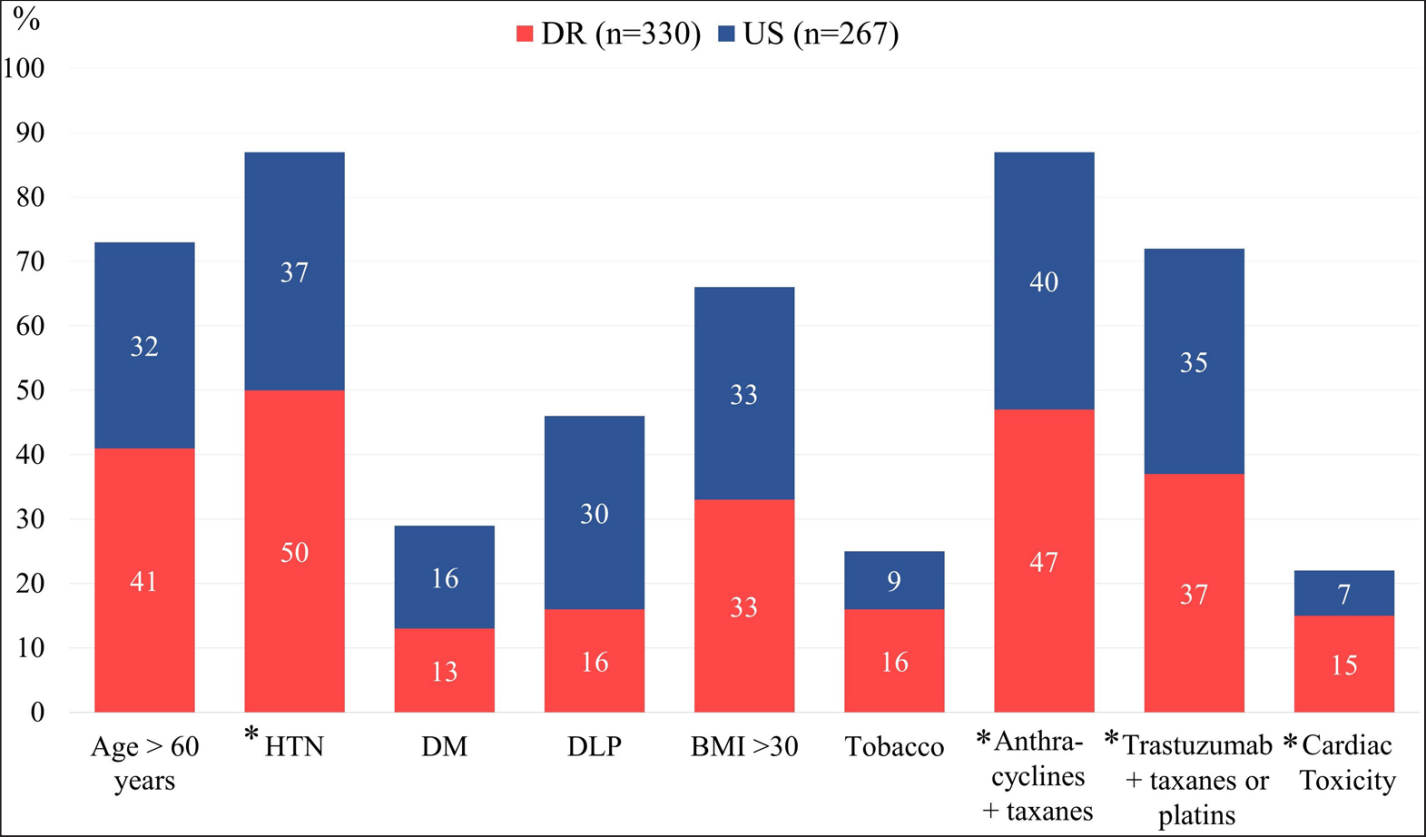
Central Illustration. Cardiovascular risk factors and rate of cancer therapy-induced cardiotoxicity. **Legend:** BMI: body mass index, DLP: dyslipidemia, DM: diabetes mellitus, DR: Dominican Republic, HTN: hypertension, US: United States. * p =< 0.001.

The overall CTx rate was 15/7% (p < 0.001): 31 (61%)/12 (60%) had a reduction on EF >10% to a value < 53% plus a drop >15% in GLS. In addition, 20 (39%)/8 (40%) had a decline in GLS only, categorized as subclinical cardiotoxicity (Figure 1: Central Illustration). Signs and symptoms of heart failure were present in 3/2% of patients, and 1/1% required HF hospitalization. Multivariate logistic regression (OR 2.24; 95% CI, 1.28–3.91; p < 0.005) did not identify age >60, HTN, DM, BMI, tobacco, or chemotherapy regimen as predictors of CTx, only DR ethnicity did (Table 2). At the first office visit, patients who developed CTx 4/0% had elevated blood pressure; otherwise, all other cardiovascular risk factors were controlled.

**Table 2:** Multivariate logistic regression analysis for cancer therapy-induced cardiotoxicity.


	ODDS RATIO	95%	CI	p

**DR/US**	2.24	1.28	3.91	<0.004

**Hypertension**	1.35	0.76	2.40	0.295

**Diabetes mellitus**	1.43	0.71	2.87	0.311

**BMI ≥ 30**	1.16	0.68	1.96	0.579

**Tobacco use**	0.97	0.46	2.02	0.937

**Age ≥ 60 years**	0.65	0.36	1.17	0.157

**Chemo: Anthracyclines + Taxanes**	1.48	0.87	2.52	0.142

**Chemo: Trastuzumab + Taxanes or Platins**	1.05	0.61	1.82	0.837


BMI: body mass index, CI: confidence interval, DR: Dominican Republic, US: United States.

Adherence to ASCO was evaluated by accounting for established pre-treatment preventive strategies, including CV evaluation and a baseline echocardiogram, with 100% compliance found in both groups (Table 3). In addition, 48/43% (p = 0.095) of patients were already on cardioprotective drugs (Angiotensin-converting enzyme inhibitors, Calcium channel blockers, Beta-blockers, Statins) before chemotherapy.

**Table 3:** Adherence to American Society of Clinical Oncology Guidelines.


RECOMMENDATIONS	DR	US	*p*

**Pre-treatment preventive strategies to reduce risk**	**n = 330 (%)**	**n = 267 (%)**	

Cardiovascular evaluation	330 (100)	267 (100)	p = 0.47

Echocardiogram	330 (100)	267 (100)	p = 0.47

Already on cardioprotective drugs	160 (48)	114 (43)	p = 0.095

**Management during cancer treatment**			

Follow-up echocardiogram	180 (54)	99 (37)	p =< 0.001

>1 echo during treatment	38 (12)	50 (19)	p = 0.009

Use of biomarkers	152 (46)	39 (18)	p =< 0.001

Developed cardiotoxicity	51 (15)	20 (7)	p =< 0.001

Cardio-oncology clinic follow up	180 (54)	99 (37)	p =< 0.001

**Monitoring after cancer treatment**			

Cardio-oncology clinic follow-up	180 (54)	99 (37)	p =< 0.001

Surveillance echocardiogram	180 (54)	99 (37)	p =< 0.001


DR: Dominican Republic, US: United States.

During cancer treatment, 54/37% (p =< 0.001) of patients were seen in the cardio-oncology clinic, and they all had follow-up echocardiograms as determined by cardiotoxicity risk according to ASCO guidelines recommendations. The remaining patients either received follow-ups at other centers or did not attend their planned visits. Follow-up studies were obtained at a mean of three months; some patients (12/19% p = 0.009) had more than one study performed if symptoms developed. There was sub-utilization of biomarkers since they were 46/18% (p =< 0.001) of the cases. After cancer treatment, all follow-up patients underwent surveillance echocardiograms and subsequent clinic visits.

## Discussion

The most important findings of this study, comparing to the US cohort are: 1) Caribbean cohort cancer patients have a similar prevalence of CV risk factors; 2) they have twice the likelihood of developing drug-induced LV dysfunction, even though many are already taking ‘cardioprotective’ therapies; and 3) local cardio-oncology programs can achieve similar rates of adherence to guidelines as in the US cohort.

We consider that cardiac evaluation based on ASCO recommendations in the Caribbean region remain suboptimal, as established cardio-oncology programs are scarce in the area. Although the published literature suggests that some progress has been made in this regard, implementation and compliance with protocols geared towards early detection of CTx is still a challenge in developing nations, often due to physician’s adherence, access to health care, and patient’s lack of awareness [7,8,9,10].

Our findings are similar to those in the limited case series published in Spanish literature [8,9,10]. In Uruguay, Camejo et al. evaluated 69 breast cancer patients treated with trastuzumab, 27% of whom developed CTx (LVEF reduction >10% to a value <55%) in a mean time of 9 months [8]. In Argentina, Santos et al. studied 888 breast cancer subjects treated with trastuzumab and detected a decline in LVEF >10% in 35% of the cohort over a mean follow-up of 48±12 months [10]. Compared to our series, the CTx rates were higher in both reports; however, the more extended follow-up period in the Santos series may have influenced their results. No data on myocardial deformation was reported in these papers. In addition to the well-recognized inaccuracies of EF measurement by 2D-echo in determining CTx, definitive comparisons cannot be made due to the lack of standardization of CTx criteria [11].

Armenian et al., found that the presence of two or more CV risk factors bestowed the highest risk of CTx (IRR 1.83–2.59) to those affected by lung or breast cancer [12]. In contrast, our results did not identify age >60 years, HTN, DM, BMI, tobacco, or chemotherapy protocols as predictors of CTx. Although the DR cohort had more hypertensive patients and the US cohort used more trastuzumab-based chemotherapy regimens, none of these parameters were identified as predictors, and the highest risk of cardiotoxicity was conferred to patient ethnic origin, that is, DR over the US. Whether these findings result from genetic predisposition or cultural or demographic variations, their true explanation rests unclear, indicating the need for further population-based and translational research.

Even though 50% of patients in both cohorts were already using ‘cardioprotective’ drugs prescribed for other reasons before initiating chemotherapy (mainly angiotensin-converting enzyme inhibitors, calcium channel blockers, beta-blockers for HTN), DR patients again had twice the likelihood of developing CTx. The benefit of these drugs in cancer populations is a topic of ongoing investigation [13,14,15,16]. Likewise, the evaluation of recovered ejection fraction in patients with cardioprotective drugs and/or detention of chemotherapy regimens was not included in this research. There was a higher use of anthracycline-based protocols in the DR cohort, however, several questions are still unanswered: if selected dose chemotherapy regimen, BMI-adjusted dose, cumulative anthracycline dose, type of cancer mutation, cancer staging, or patient’s adherence to cardioprotective drug therapy influenced the resulting LV dysfunction. Risk stratification protocols and preventive strategies need to be pursued between oncology and cardiology services in order to reduce the rate of cardiotoxicity in the DR cohort.

Dominican women included in this series had a higher prevalence of CV risk factors than men, an interesting find since recent local surveys in the DR have shown otherwise [5]. Additionally, the overall prevalence of such risk factors in our cohort was twice as high as reported figures in the US cohort; again, this underscores the need for population-based initiatives aimed at risk factors modification and control [17].

Lastly, although biomarkers and echocardiography utilization varied between the two centers, compliance with ASCO recommendations was similar, particularly in outpatient follow-up. In the DR cohort, fewer patients underwent echocardiographic monitoring during follow-up, even as the guidelines were strictly followed.

Cardiac troponin plasma concentrations can predict CTx in patients treated with anthracyclines and trastuzumab, and NT-proBNP has been recognized as an independent predictor of all-cause mortality in cancer patients [2,18,19]. Although the importance of measuring biomarkers periodically cannot be overemphasized, it remains uncertain if their use in conjunction with echocardiography constitutes a cost-effective approach in nations with limited resources.

We recognize that as most referrals came from within the institutions involved in the study, this may represent a selection bias since treating physicians were theoretically in more contact with the investigators, threatening the generalizability of the findings. The analysis of other severe adverse events like coronary artery disease, arrhythmia, or venous thromboembolism induced by chemotherapy were out of the scope of this investigation. Similarly, regimen doses, cumulative anthracyclines dose, and the association of cancer types and treatment protocols with cardiotoxicity were variables not included in this study. We understand our results comprise two specific cohorts of both countries; therefore, the findings cannot be generalized to the reality of other centers or nations in America. We believe this study traces a road map of what is being done in the region’s day-to-day world of clinical practice, rather than instructing or designing specific quality improvement initiatives at this junction. Its main strength is closing the gap in data on the regional CV profile of oncological patients in the Caribbean. Finally, although the data were obtained in the DR and therefore possibly considered not applicable to the rest of the Caribbean region, we believe that given the socioeconomic, geographical, and ethnic similarities among the rest of the nations of the area (except perhaps Cuba, with a fully socialized health care system), our results may reflect the current situation in neighboring countries.

## Conclusion

Compared to the US cohort, the Caribbean cohort of cancer patients has similar rates of CV risk factors but a higher likelihood of developing drug-induced LV dysfunction. Programs’ compliance with ASCO guidelines was equivalent. These findings highlight the need for the Latin-American scientific medical community to join forces toward achieving common goals in the fight against chemotherapy mediated CTx. We believe that the next natural step in this endeavor should be the implementation of local and regional multidisciplinary, multicenter, structured protocols and measuring their future impact on a larger scale We hope that this initiative contributes to such planning and that it leads to system-wide policies beneficial to the cardio-oncological population.

## Author Notes

All authors take responsibility for all aspects of the reliability and freedom from bias of the data presented and their discussed interpretation.
